# The development process of a regional dementia strategy: a Delphi study and deliberative dialogues

**DOI:** 10.1186/s12913-026-15595-8

**Published:** 2026-09-22

**Authors:** Christine Schiller, Gabriele Meyer, Anja Bieber

**Affiliations:** Institute of Health, Midwifery and Nursing Science, Medical Faculty of Martin Luther University Halle-Wittenberg, University Medicine Halle, Halle (Saale), Germany

**Keywords:** Dementia, Regional health policy, Dementia Strategy, Participatory research, Delphi method, Interest-holder engagement

## Abstract

**Background:**

Over the past decade, numerous countries have adopted national dementia strategies to address the growing societal impact of dementia. In Germany, six federal states have regional strategies to address local demographic and structural challenges, such as those in Saxony-Anhalt, Germany’s fastest shrinking and ageing federal state with the highest dementia prevalence. In 2022, the State Competence Center for Dementia Saxony-Anhalt was established to develop a comprehensive regional Dementia Strategy. The aim of this study was to generate consensus on the objectives required to ensure adequate care and improve the quality of life of people living with dementia and their families in Saxony-Anhalt. To achieve this, the study used a participatory approach to identify and prioritize strategic objectives for the regional Dementia Strategy.

**Methods:**

A mixed-method, two-phase participatory design was employed. First, in a two-round Delphi process ratings from nine interest-holder groups were collected, including people living with dementia, informal caregivers, professionals, policymakers, and civil society representatives. Second, deliberative dialogues explored selected non-consensus objectives. Consolidated objectives were thematically grouped and aligned with state ministries to ensure policy feasibility.

**Results:**

In total, 406 participants completed round 1, with 262 participants completing round 2; 18 participants attended deliberative dialogues. Of 88 initial objectives, 47 reached consensus in round 1, 21 more in round 2, and seven were refined and validated through deliberative dialogues. The remaining 26 non-consensus items were thematically aggregated into broader strategic objectives. After ministerial alignment, the final Dementia Strategy comprises 40 strategic objectives across four main topics: social participation, medical and nursing care, support for people with dementia and informal caregivers, and promotion of dementia research. Participants also identified structural challenges, including service shortages, workforce limitations, bureaucratic barriers, and caregiver burden. These were documented as contextual factors relevant to implementation.

**Conclusion:**

This study demonstrates the feasibility of a structured participatory approach for regional dementia strategy development. The resulting strategy provides a foundation for future action, while successful implementation and the development of an implementation strategy will be essential to translate agreed objectives into practice. The approach may serve as a transferable model for evidence-informed and participatory health policy development.

**Supplementary Information:**

The online version contains supplementary material available at https://doi.org/10.1186/s12913-026-15595-8.

## Background

Over the past decade, many countries have adopted national dementia strategies to address the growing societal and health system impact of dementia [1]. Six out of the 16 German federal states have regional dementia strategies or action plans to adapt national frameworks to local demographic and structural conditions [2]. In 2020, Germany adopted its National Dementia Strategy, providing a framework to guide national efforts to improve care, support, and awareness for people living with dementia and their families. The National Dementia Strategy was developed through a participatory process involving federal ministries, researchers, healthcare providers, advocacy organizations, people living with dementia, family caregivers, and other civil society interest-holders. This collaborative approach resulted in a broad set of strategic objectives and measures addressing dementia care, support, and social participation [3].

Comparative analyses of national and regional dementia strategies show that a variety of approaches have been used to develop dementia policies, including consultations with interest-holders, expert working groups, workshops, and other collaborative planning processes. While many strategy documents describe the actors involved and the resulting priorities or action areas, the extent to which the underlying processes for identifying, prioritizing, and translating objectives into strategic goals are reported varies considerably [4, 5]. Transparent reporting of strategy development processes may therefore contribute to the transferability, legitimacy, and implementation of dementia policies across different contexts.

Dementia is associated with high health and long-term care costs, workforce shortages in geriatric and nursing care, and substantial psychosocial strain among families [6, 7]. Moreover, differences in service availability and coordination across regions contribute to inequities in diagnosis, treatment, and support [8, 9]. Addressing dementia therefore requires an integrated approach that combines medical, social, and policy perspectives at both national and regional levels [10, 11].

Regional initiatives build upon this national framework to address local demographic and healthcare challenges. Saxony-Anhalt is currently the fastest shrinking and ageing federal state in Germany [12]. It already has the highest proportion of people living with dementia nationwide [13]. Moreover, the prevalence of dementia varies considerably between districts [14], creating uneven care needs and access to services. Furthermore, compared with the German average, Saxony-Anhalt has a substantially lower population density [15], which may contribute to longer travel distances and challenges in ensuring equitable access to dementia-related services and support, particularly in rural areas. These demographic and structural characteristics pose specific challenges for the region and underline the need for a tailored, evidence-based approach.

While the national strategy provides overarching goals and policy recommendations, the planning and delivery of health and social care services are largely shaped at regional and local levels. Regional dementia strategies therefore play an important role in translating national objectives into actions that reflect local demographic, structural, and organizational conditions. In addition, involving regional interest-holders early in the strategy development process can strengthen ownership, foster collaboration across sectors, and support the subsequent implementation of strategic objectives. These considerations are particularly relevant in Saxony-Anhalt, given the federal state’s ageing and shrinking population, high dementia prevalence, and regional variation in care needs and service provision.

To address these challenges, the State Competence Center for Dementia Saxony-Anhalt (Landeskompetenzzentrum Demenz Sachsen-Anhalt) was established in 2022 to identify regional needs, strengthen care structures, and support sustainable solutions. A central task of the Center is the development of a comprehensive Dementia Strategy for the federal state. The strategy aims to improve the quality of life of people living with dementia and their families, enhance care coordination, and promote dementia-friendly communities across the state.

Taken together, these observations highlight the need for structured and transparent approaches that systematically integrate the perspectives of diverse interest-holders during the early phases of dementia strategy development. In Germany, existing regional dementia initiatives have so far remained largely descriptive and vary considerably in scope, with limited use of structured, evidence-based, or participatory methods. This gap highlights the importance of using structured, evidence-informed, and participatory approaches to identify and prioritize regional objectives that genuinely reflect the needs and experiences of people affected by dementia.

Evidence shows that participatory methods, such as Delphi processes and deliberative dialogues, can facilitate the identification of shared priorities, increase transparency, and strengthen the legitimacy of policy outcomes [16, 17]. These participatory approaches are particularly suitable for complex public health issues like dementia, which require collaboration across professional, institutional, and societal boundaries.

The development process was conceptually informed by policy development frameworks that emphasize evidence-informed decision-making driven by diverse interest-holders [18]. Applying these principles ensures that the resulting strategy is both scientifically grounded and practically relevant for regional health planning.

The development of the Dementia Strategy for the federal state follows two main steps: first, identifying and prioritizing objectives for the federal state of Saxony-Anhalt, based on the German National Dementia Strategy [3] and international recommendations [10, 19]; and second, developing concrete implementation strategies to ensure sustainable impact.

Therefore, the aim of this study was to generate consensus on the objectives required to ensure adequate care and improve the quality of life of people living with dementia and their families in Saxony-Anhalt. This paper describes the methodological approach used in the first step, identifying and prioritizing key objectives for the Dementia Strategy, using a Delphi consensus process complemented by deliberative dialogues.

## Methods

### Study design

This study used a two-phase participatory design to identify and prioritize key objectives for the Dementia Strategy of Saxony-Anhalt. Phase 1 consisted of a two-round Delphi consensus process [20, 21] to identify and prioritize objectives proposed for the strategy. Phase 2 comprised deliberative dialogues [17, 22, 23], thematic synthesis of Delphi and dialogue findings, and a policy consultation process with relevant state ministries. This mixed-method approach combined quantitative consensus measurement with qualitative exploration of divergent perspectives and subsequent policy validation.

### Phase 1: Delphi consensus process

#### Participants and recruitment

Based on the thematic fields and actor groups outlined in the German National Dementia Strategy, relevant groups of interest-holders were defined prior to recruitment to ensure balanced representation of all perspectives involved in dementia care and policy. Eligible participants had to live or work in Saxony-Anhalt and to have direct or indirect contact with people living with dementia in their professional or everyday contexts. Individuals in decision-making roles relevant to dementia care and support were also included.

Participants were assigned to nine interest-holder groups: (1) people living with dementia, (2) informal caregivers, (3) health and social care professionals, (4) payers and policymakers, (5) public administration representatives, (6) members of the education sector, (7) dementia researchers, (8) volunteers engaged in dementia-related activities, and (9) civil society representatives.

People living with dementia were included only if they were able to provide informed consent, assessed individually based on their understanding, reasoning, and decision-making capacity. Potential participants were recruited through established organizational networks, ministries, umbrella organizations, and dementia-related associations.

#### Data collection

The Delphi survey was conducted in two rounds between June 2024 and November 2024. It was designed to collect and prioritize expert assessments of proposed objectives for the regional Dementia Strategy. Participants were pseudonymized and surveyed using a standardized questionnaire administered either online via LimeSurvey (version 6.15.21; LimeSurvey GmbH, Hamburg, Germany) or on paper.

The questionnaire covered four main topics: (1) social participation, (2) medical and nursing care, (3) support for people living with dementia and their informal caregivers, and (4) promotion of dementia research. The Delphi questionnaires used in both rounds were specifically developed for this study (see Additional file 1 and Additional file 2). The questionnaire items corresponded to proposed strategic objectives and were formulated as statements to be rated regarding their importance for the regional Dementia Strategy.

The initial Delphi questionnaire was developed through a structured multi-step process based on policy documents and the expertise of the interdisciplinary project team. The German National Dementia Strategy [3] served as the primary source, providing the overall thematic structure and forming the basis for the development of the initial objectives. To ensure consistency with international recommendations, the WHO Global Action Plan on the Public Health Response to Dementia 2017–2025 [10] was reviewed as an overarching conceptual framework. In addition, existing regional dementia strategies and action plans from several German federal states were consulted to identify additional implementation approaches and region-specific aspects relevant to the development of a regional Dementia Strategy for Saxony-Anhalt [24, 25].

In a first step, objectives addressing the care, support, participation, and quality of life of people living with dementia and their informal caregivers were identified and synthesized. Overlapping objectives were consolidated, duplicates were removed, and the wording was harmonized to create a coherent set of objectives.

In a second step, the resulting objectives were critically reviewed by the project team regarding their relevance to the regional context of Saxony-Anhalt. Where necessary, objectives derived from the German National Dementia Strategy were adapted to reflect regional structures, responsibilities, and implementation opportunities. Where relevant aspects were considered insufficiently represented, additional objectives were formulated during the synthesis process based on nursing science expertise and regional needs.

Finally, all objectives were categorized into four thematic areas reflecting the structure of the German National Dementia Strategy: (1) social participation, (2) medical and nursing care, (3) support for people living with dementia and their informal caregivers, and (4) dementia research. This process resulted in the initial Delphi questionnaire comprising 88 objectives. The detailed overview of the initial objectives, their correspondence with the German National Dementia Strategy and the WHO Global Action Plan, and adaptations or additions made during the synthesis process is provided in Appendix 1.

The questionnaire was pilot-tested with two representatives of key interest-holder groups, including one person living with dementia and one informal caregiver. The pilot testing focused on the clarity, accessibility, and comprehensibility of the items, as well as the appropriateness of terminology and response formats. Based on the feedback received, wording and structure of several items were refined to improve accessibility before the questionnaire was administered in the first Delphi round.

In both Delphi rounds, participants rated the importance of each proposed objective on a seven-point Likert scale (1 = “not important” to 7 = “extremely important”). Technical terms were explained in an accompanying glossary. Respondents could also propose additional objectives or comment on unclear items.

Following analysis of round 1, participants received a revised questionnaire for round 2 containing (a) all objectives that had not reached consensus and (b) new objectives proposed in round 1. Participants also received summarized feedback from the previous round, enabling them to reconsider or confirm their earlier ratings. Each round remained open for approximately eight weeks, and one reminder was sent to all invited participants.

#### Data analysis

Quantitative data were analyzed using statistical aggregation of group responses in LimeSurvey. Consensus was defined according to predefined criteria in the unpublished study protocol: a median rating ≥ 6, an interquartile range (IQR) ≤ 0.5, and at least 75% of responses within two adjacent values. When these criteria were met, an objective was considered “agreed upon” and included in the Dementia Strategy. No differential weighting of specific participant groups was applied. All responses were treated equally in the aggregation process, and consensus was determined across the total sample based on the predefined statistical criteria.

Open-text responses were analyzed using qualitative content analysis in MAXQDA (version 24; VERBI Software GmbH, Berlin, Germany). Responses were initially coded inductively to identify recurring themes and patterns. Codes were discussed iteratively within the research team and refined, and any discrepancies were resolved through consensus. Additional objectives suggested by participants were categorized under emerging themes and, where appropriate, incorporated into the second Delphi round. Following completion of round 1, summarized quantitative results and qualitative feedback were provided to participants as part of the second-round questionnaire, enabling them to reconsider their initial ratings in light of the group responses. Objectives that did not reach consensus after round 2 were carried forward to Phase 2 for further deliberation and thematic synthesis.

### Phase 2: deliberative dialogues, thematic synthesis and policy consultation

#### Participants and recruitment

Workshop participants were mainly drawn from the Delphi panel. Additional purposive recruitment was undertaken to ensure representation of underrepresented perspectives, particularly those of people living with dementia.

#### Data collection

After completion of the Delphi survey, three deliberative dialogues were conducted to further discuss objectives that had not achieved consensus. Three workshops were organized to allow manageable group sizes and to ensure broad representation of perspectives while maintaining the interactive character required for deliberative exchange. The dialogues followed a deliberative dialogue approach, promoting balanced exchange and collective reflection among participants [9–11]. A structured discussion guide, including predefined questions and a standardized workshop procedure, was developed specifically for the deliberative dialogues (see Additional file 3). Each dialogue focused on the same set of non-consensus objectives to ensure comparability and explored underlying reasons for disagreement.

Objectives were selected for deliberative discussion based on two main criteria: their perceived need for conceptual clarification and their normative or interpretative complexity, where multiple perspectives could lead to differing priorities or interpretations. In particular, items related to awareness-raising and societal sensitization were prioritized as a cross-cutting theme, since these objectives were identified as conceptually complex and highly relevant across several action areas. Objectives that were sufficiently clear in content but had not reached consensus were instead thematically grouped and integrated into broader higher-level strategic objectives during the subsequent synthesis phase. The deliberative dialogues were held as half-day events with two 90-minute sessions, each involving up to ten participants. All participants received a summary of the Delphi results in advance to establish a common knowledge base.

#### Data analysis

Discussions were documented, summarized, and returned to participants for validation. Workshop data were analyzed descriptively and qualitatively to identify whether remaining disagreements could be resolved or translated into coexisting, well-reasoned positions. Objectives validated through deliberative dialogues were subsequently included in the thematic synthesis together with objectives that had achieved consensus during the Delphi process.

#### Thematic synthesis of Delphi and dialogue findings

Following completion of the Delphi process and deliberative dialogues, all objectives were synthesized through an iterative thematic aggregation process. Objectives that achieved consensus in the Delphi process, as well as objectives validated through deliberative dialogues, were reviewed and grouped according to thematic similarity, strategic relevance, and their contribution to the overall objectives of the Dementia Strategy. A detailed overview of all sub-objectives, including their Delphi ratings, workshop discussions, interministerial consultation, and final status, is presented in Appendix 1. This table allows for transparent tracking of the decision pathway for each objective and shows which objectives were included through consensus, workshop validation, or thematic aggregation.

To create a manageable and policy-relevant strategy document, overlapping or closely related objectives were merged while preserving their original content and intent. Objectives that had not achieved consensus but were considered sufficiently clear in content were reviewed and, where appropriate, integrated into broader higher-level strategic objectives.

Importantly, thematic aggregation did not imply retrospective consensus on individual objectives. Information regarding the lack of consensus was retained throughout the documentation and interpretation of findings to ensure transparency regarding divergent perspectives.

The resulting synthesis produced a consolidated set of strategic objectives organized into overarching thematic domains aligned with the main topics of the National Dementia Strategy and international recommendations.

#### Policy consultation with state ministries

The consolidated draft strategy was submitted to relevant state ministries of Saxony-Anhalt for consultation. This alignment process aimed to ensure political feasibility, administrative coherence, and compatibility with existing state-level strategies and legal frameworks.

Feedback from the ministries was reviewed by the project team and incorporated where appropriate. Objectives derived from the Delphi process and deliberative dialogues were not modified, excluded, or reprioritized as part of the consultation process.

Following the policy consultation, several rounds of internal revision were undertaken to improve the clarity, consistency, and readability of the strategy document. These revisions did not alter the agreed objectives or priorities but focused on refining the presentation and formulation of the strategy. The resulting document constituted the final Dementia Strategy of Saxony-Anhalt.

## Results

### Phase 1: Delphi consensus process

#### Participants

In the first Delphi round, a total of 406 participants completed the questionnaire. Participants represented all predefined interest-holder groups and reflected a wide range of professional backgrounds and levels of contact with people living with dementia (Table 1). Participants were recruited from all regions of the federal state and completed the survey either online (*n* = 369) or using the paper-based version (*n* = 37).

In the second Delphi round, 262 participants took part. The composition of participants remained comparable to round 1 with regard to interest-holder groups, regional distribution, and mode of participation (online vs. paper-based).

**Table 1:** Characteristics of participants in the delphi survey (rounds 1 and 2) and deliberative dialogues data are presented as number (percentage) unless otherwise indicated

Characteristic	Delphi Round 1 406 (100,0%)	Delphi Round 2 262 (64,5%)	Deliberative Dialogues 18
**Type of respondent**			
Individual	287 (70,7%)	196 (74,8%)	18 (100,0%)
Institution/ organization	119 (29,3%)	66 (25,2%)	0 (0,0%)
**Professional background** ^1^			
Social and healthcare professionals	246 (60,6%)	142 (54,2%)	3 (16,7%)
Payers and policymakers	13 (3,2%)	7 (2,7%)	2 (11,1%)
Public administration representatives	83 (20,4%)	45 (17,2%)	3 (16,7%)
Members of the education sector	42 (10,3%)	29 (11,1%)	2 (11,1%)
Dementia researchers	8 (2,0%)	8 (3,1%)	2 (11,1%)
Volunteers engaged in dementia-related activities	18 (4,4%)	11 (4,2%)	1 (5,6%)
Representatives of civil society organizations	5 (1,2%)	3 (1,1%)	0 (0,0%)
Other professional backgrounds	28 (6,9%)	19 (7,3%)	0 (0,0%)
Not working	34 (8,4%)	34 (13,0%)	---
**Relationship to people living with dementia** ^1^			
Person with dementia	1 (0,2%)	2^2^ (0,8%)	2 (11,1%)
Informal caregiver	147 (36,2%)	102 (38,9%)	3 (16,7%)
Professionally in contact with people with dementia/ their informal caregiver	215 (53,0%)	125 (47,7%)	---
Professionally responsible for care/ medical framework (e.g., health insurance, policy makers)	46 (11,3%)	31 (11,8%)	---
No direct contact with people with dementia/ their informal caregiver	51 (12,6%)	38 (14,5%)	---

¹ *Multiple responses possible. Percentages are calculated based on the total number of respondents per round/workshop; sums may exceed 100%*

^*2*^ *One participant received a diagnosis of dementia during the ongoing Delphi process*

#### Delphi findings

**Fig. 1:**
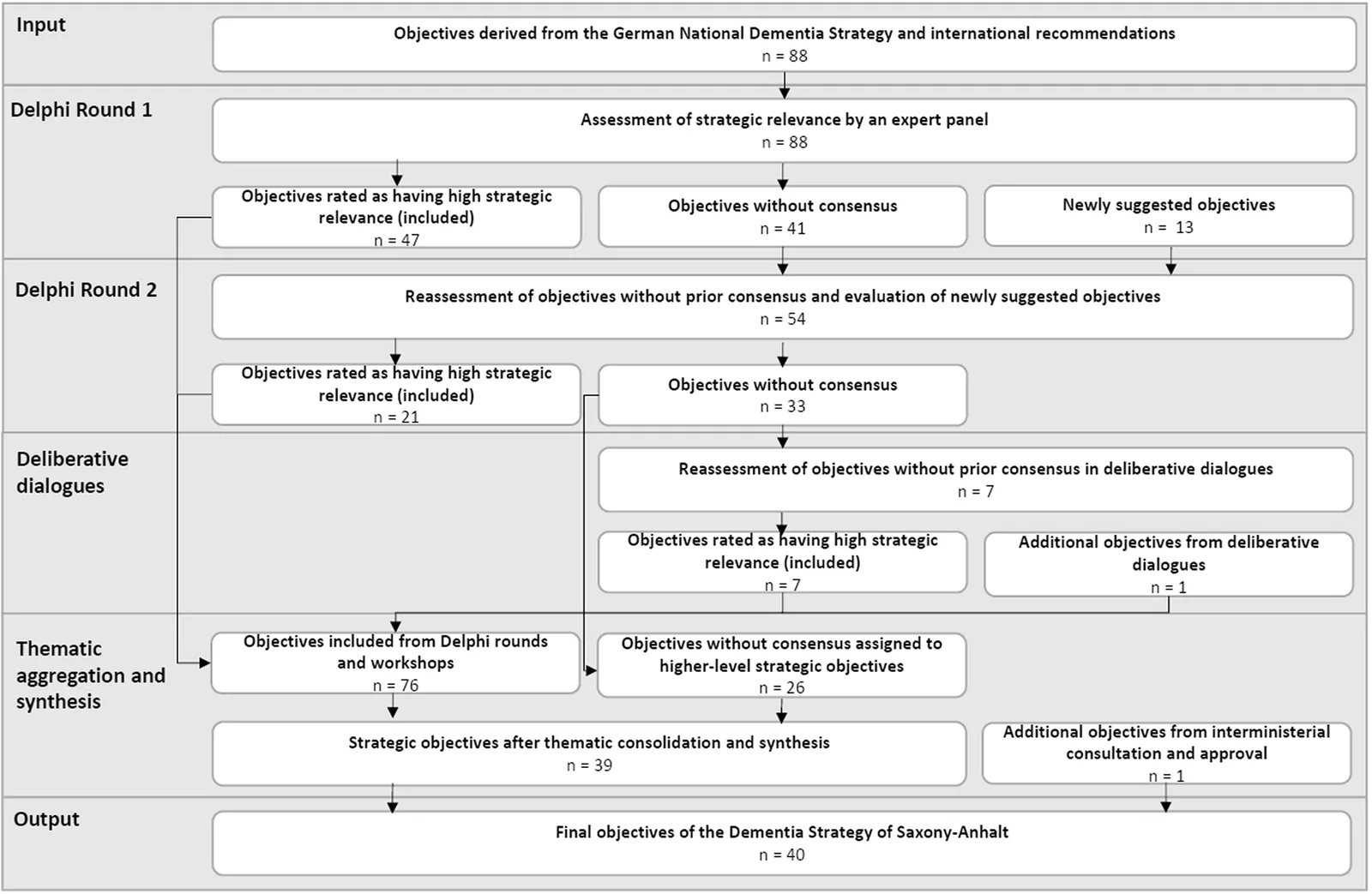
Flow diagram of Delphi rounds and deliberative dialogues

A total of 88 initial objectives, developed through the synthesis process described above, were assessed across four predefined main topics. Of these, 47 objectives reached consensus and were directly included in the Dementia Strategy, while 41 objectives did not reach consensus (Fig. 1). In addition, participants proposed 13 additional objectives through open-text responses.

Beyond these additional objectives, respondents suggested structural challenges specific to the federal state, including limited service availability in rural areas, workforce shortages, bureaucratic barriers, insufficient cross-sectoral coordination, and financial burden for informal caregivers. These challenges were documented as contextual constraints relevant for implementation, but were not formulated as strategic objectives.

Round 2 included the 41 objectives without prior consensus from round 1 as well as the 13 newly proposed objectives, resulting in a total of 54 objectives being reassessed. Of these, 21 objectives reached consensus and were rated as having high strategic relevance. The remaining 33 objectives did not reach the predefined consensus threshold (Fig. 1).

After two Delphi rounds, not all items had reached consensus. Objectives without consensus were therefore handled using two complementary approaches. A comprehensive overview of all objectives and their progression through the subsequent stages of the strategy development process is provided in Appendix 1.

### Phase 2: Deliberative dialogues, thematic synthesis and policy consultation

#### Deliberative dialogues

The 33 non-consensus objectives were addressed using two complementary approaches. First, seven strategically relevant objectives were discussed and refined in deliberative dialogues involving participants from the Delphi survey. Following these discussions, all seven objectives were assessed as strategically relevant and included in the strategy.

#### Thematic synthesis of Delphi and dialogue findings

Second, the remaining 26 objectives, many of which addressed specific operational aspects, were thematically aggregated into broader strategic objectives during the synthesis phase. The lack of consensus at the individual item level was explicitly retained in the documentation and did not imply agreement. Appendix 1 provides a detailed overview of how all non-consensus objectives were handled.

No objectives were excluded solely because they lacked majority support; all proposed objectives were either refined through deliberation or integrated into higher-level strategic objectives to preserve the breadth of perspectives expressed.

#### Policy consultation with state ministries

Based on the combined results of the Delphi survey and the deliberative dialogues, objectives were thematically aggregated and synthesised, resulting in 39 strategic objectives. The draft strategy was then submitted to seven state ministries of the federal state for formal review and written feedback to ensure consistency with existing policy frameworks and administrative responsibilities.

During this interministerial consultation, feedback primarily concerned alignment with existing state-level policy frameworks and ongoing initiatives. One additional objective – “Information and training programs for the prevention of dementia are available” – was proposed and incorporated into the strategy. No objectives derived from the participatory process were modified, excluded, or reprioritized.

Following ministerial approval, the final Dementia Strategy of the federal state comprises 40 objectives (Fig. 1; Appendix 1).

### Final dementia strategy

To summarize the substantive content of the strategy, the 40 strategic objectives are structured across four main topics: (1) social participation of people living with dementia, (2) nursing and medical care, (3) support for people living with dementia and their informal caregivers, and (4) dementia research.

Within social participation, the strategy promotes dementia-friendly municipalities and neighbourhoods, strengthened local networks, improved living environments, cultural and leisure participation, digital inclusion, and structured awareness-raising measures.

Under nursing and medical care, priorities include dementia-friendly organization of care facilities, guideline-based and coordinated treatment, expansion of therapeutic services, improved hospital care, and the development of alternative dementia-friendly care models.

The main topic support for people living with dementia and their informal caregivers encompasses community-based counselling, legal support structures, reconciliation of caregiving and employment, voluntary and self-help initiatives, prevention and rehabilitation programs, and dementia-friendly palliative care.

Within dementia research, the strategy promotes the development and implementation of evidence-based concepts, greater attention to everyday life contexts, stronger collaboration between research and practice, the establishment of a statewide research network, and the meaningful involvement of people living with dementia and their informal caregivers in research projects relevant to them.

## Discussion

This study applied a participatory, mixed-method approach to develop the Dementia Strategy at the level of a federal state in Germany, combining a two-round Delphi process with deliberative dialogues and a subsequent policy alignment step. The findings illustrate both the potential and the inherent challenges of consensus-oriented approaches in regional health policy development, particularly in complex fields such as dementia care.

### Participatory approaches in dementia strategy development

Comparative analyses of national and regional dementia strategies indicate that participatory approaches are increasingly employed in dementia policy development, with the involvement of people living with dementia, their relatives, and diverse interest-holders regarded as crucial for ensuring practical relevance, legitimacy, and acceptance of resulting strategies [4, 26]. Against this background, the present study operationalizes participatory strategy development through a structured combination of consensus measurement and deliberative dialogues, thereby translating international recommendations on inclusive dementia policy-making into regional context.

The Delphi process enabled a structured and transparent elicitation and quantification of expert assessments across heterogeneous interest-holder groups. Such approaches are well established in health services research as a means of structuring expert knowledge and identifying areas of agreement in complex decision-making environments [21, 27]. However, published descriptions of national and regional dementia strategies rarely report the systematic use of formal consensus techniques such as Delphi processes during early priority-setting phases [4, 11]. Strategy development is often described in broad participatory terms, but methodological transparency regarding how priorities are identified and aggregated remains limited. In this respect, the present study contributes a structured and explicitly documented consensus procedure to the field of dementia policy development. Complementing this quantitative component, the deliberative dialogues provided space for in-depth qualitative reflection, clarification of underlying values, and the integration of non-professional perspectives that are often insufficiently captured through survey-based methods alone.

Across two Delphi rounds, a substantial proportion of objectives reached consensus, indicating a high degree of shared understanding regarding strategic priorities for dementia care in the federal state. The relatively stable composition of participants across rounds supports the robustness of these findings. At the same time, a considerable number of objectives did not reach the predefined consensus threshold, underscoring the complexity of dementia care and the persistence of divergent perspectives, particularly with respect to operational and structural aspects. Comparative analyses of national dementia plans similarly demonstrate considerable variation in strategic priorities, preventive approaches, and role allocations, reflecting the multidimensional and contested nature of dementia policy-making [11, 28]. Such findings suggest that disagreement on specific objectives does not necessarily indicate methodological weakness but rather mirror broader structural and normative tensions within dementia care systems.

### Handling of non-consensus objectives

Rather than being interpreted as a methodological limitation, the absence of consensus for certain objectives was understood as reflecting legitimate and enduring differences in priorities, values, and interpretations of responsibility. Appendix 1 details which objectives were refined and included through deliberative dialogues and which were thematically aggregated into higher-level strategic objectives. This transparent documentation preserves the traceability of decision-making and highlights the persistence of divergent perspectives. Previous studies have emphasised that non-consensus in Delphi processes can yield valuable insights into contested areas and should not automatically be resolved or eliminated [29, 30].

Deliberative dialogues enabled a subset of these contested objectives to be explored in greater depth and, where possible, refined. However, only a limited number of non-consensus objectives could be addressed in this format. For the remaining objectives, thematic aggregation into higher-level strategic objectives was employed. Importantly, the lack of consensus at the individual item level was explicitly retained in the documentation and interpretation of results and was not interpreted as agreement. This distinction is essential to avoid overstating consensus and to preserve transparency regarding unresolved issues [20, 31].

An additional feature of the study was the consultation with state ministries following completion of the participatory process. While the involvement of policy actors may raise concerns regarding potential influence on stakeholder-derived priorities, the consultation was deliberately conducted only after the Delphi process and deliberative dialogues had been completed. Importantly, objectives identified through the participatory process were neither modified nor reprioritized. The consultation primarily served to ensure policy feasibility and alignment with existing state-level frameworks and resulted in the addition of a single objective related to dementia prevention.

### Inclusion of people living with dementia: challenges and strengths

The limited number of people living with dementia participating in this study (*n* = 4) highlights a central methodological challenge in participatory dementia research. As shown by Bühler et al. [26], many participatory formats implicitly rely on verbal communication skills and therefore tend to include primarily people with mild dementia. Meaningful participation of people living with dementia requires targeted methodological adaptations, such as preparatory materials, skilled facilitation, and flexible or multi-stage formats [26].

At the same time, the study demonstrates an important methodological strength. People living with dementia and informal caregivers were involved from the earliest stage of the research process, including the pilot testing of the Delphi questionnaire prior to the first survey round. This early involvement informed refinements in wording, structure, and terminology and contributed to improving the accessibility and comprehensibility of the survey instrument. Engaging people living with dementia at the instrument development stage, rather than solely as respondents, reflects principles of inclusive and participatory research and strengthens the relevance of the findings [26, 32].

The strong involvement of professional actors represents an additional consideration when interpreting the results. While professional expertise is essential for policy development, it may also lead to an overrepresentation of organisational and system-level priorities at the expense of everyday needs and experiential knowledge. Previous participatory health research has described similar imbalances, noting that institutional actors often have greater resources, familiarity with policy language, and influence within structured decision-making formats. Future strategy development processes should therefore consider measures to strengthen the participation of people living with dementia and informal caregivers, for example through targeted recruitment strategies, adapted communication formats, facilitated small-group settings, preparatory support, or weighting mechanisms that ensure experiential perspectives are not overshadowed by professional interests [26, 33, 34].

### Structural challenges beyond strategic objectives

Beyond the formulation of strategic objectives, participants of the Delphi survey identified several structural challenges that complicate the care and support of people living with dementia and their relatives, including limited service availability in rural areas, workforce shortages, bureaucratic barriers, insufficient cross-sectoral coordination, and financial burdens for informal caregivers. Comparable structural constraints have also been reported in evaluations of other regional dementia plans, where the translation of strategic objectives into practice has been shown to depend heavily on available resources, institutional coordination, and workforce capacity [24, 25, 35].

Experiences from the implementation of the German National Dementia Strategy further illustrate that comprehensive goal-setting alone does not guarantee effective translation into practice. Monitoring reports document the need for continuous coordination, structured reporting mechanisms, and iterative adaptation of governance procedures during implementation [36]. These findings suggest that dementia strategies operate within complex health and social care systems in which structural constraints significantly shape feasibility and sustainability.

In light of these implementation experiences, the structural challenges identified in the present study should not be interpreted merely as contextual remarks but as integral determinants of implementation success. Explicit acknowledgment of such constraints may support more realistic implementation planning and aligns with broader experiences in dementia policy development, where adaptive governance arrangements and sustained intersectoral collaboration have proven essential for translating strategic intent into sustainable care structures.

### Content-related priorities of the Dementia Strategy

The distribution of objectives across the four main topics reflects an integrated understanding of dementia that aligns with international policy frameworks. The strong emphasis on dementia-friendly municipalities, awareness-raising, and community-level structures corresponds with the public health orientation of the WHO Global Action Plan on the Public Health Response to Dementia 2017–2025, which highlights community inclusion and dementia-friendly societies as key priorities [10]. Comparative analyses of national dementia plans similarly identify social participation and public awareness as central strategic pillars [11].

The focus on dementia-friendly organization of care facilities, guideline-based medical treatment, and cross-sectoral coordination further reflects international calls for quality improvement and service integration in dementia care [10, 37]. OECD analyses emphasize that improving coordination between primary care, specialist services, and long-term care is essential to address fragmentation in ageing health systems [1]. By combining structural quality standards with measures that strengthen intersectoral collaboration, the strategy operationalizes these recommendations within a regional governance context.

Support for informal caregivers and the involvement of people living with dementia represent central cross-cutting themes within the strategy, reflected across multiple main topics and strategic objectives. This prioritization is consistent with extensive evidence demonstrating the substantial economic and psychosocial burden borne by informal caregivers across Europe [6, 7]. At the same time, growing research highlights the importance of actively involving people living with dementia in research, policy development, and service design to ensure that their lived experiences are adequately reflected [38]. International policy frameworks therefore highlight both caregiver support—such as rehabilitation and the reconciliation of caregiving and employment—and the participation and empowerment of people living with dementia as essential components of sustainable dementia care systems [1, 10].

Finally, the inclusion of a dedicated main topic on dementia research reflects growing international recognition of the importance of strengthening evidence generation, knowledge translation, and collaboration between research and practice [1, 10]. By integrating research networking and knowledge transfer into the strategy, evidence production and implementation are treated as interconnected processes rather than separate domains.

Taken together, these priorities demonstrate that the strategy is not limited to administrative planning but resonates with broader international developments in dementia policy. At the same time, the emphasis on rural service gaps, local networks, and workforce constraints illustrates how global policy principles must be adapted to specific regional contexts.

### Regional adaptation of the National Dementia Strategy

While the overall structure of the Dementia Strategy of Saxony-Anhalt closely aligns with the four action fields of the German National Dementia Strategy, several differences emerged [3]. Rather than creating an entirely new strategic framework, the regional strategy translates national objectives into priorities that reflect the demographic, structural, and organizational characteristics of Saxony-Anhalt.

Furthermore, the participatory approach allowed regional interest-holders to engage with the objectives of the National Dementia Strategy and to identify those considered most relevant within the regional context. This process contributed to a stronger sense of ownership and helped translate national priorities into a strategy that reflected regional perspectives and needs.

Particular emphasis was placed on strengthening regional dementia networks, improving access to therapeutic services, and enhancing the transfer of research findings into routine care through closer collaboration between research and practice. These priorities were identified as especially relevant by regional interest-holders and therefore received greater prominence within the regional strategy.

At the same time, the strategy focuses on objectives that can realistically be addressed within the competencies of regional actors. Consequently, objectives that depend primarily on federal legislation, national financing mechanisms, social insurance regulations, workforce policies, or nationwide research infrastructures are largely absent. The resulting strategy therefore complements the German National Dementia Strategy by operationalizing national objectives in a regionally relevant and implementable manner.

### Implications for future dementia policy development

Building on the developed Dementia Strategy for the federal state, the next step will be the elaboration of an implementation concept that enables participatory planning and execution of concrete measures. Extending participatory approaches beyond strategy development into implementation phases may help to revisit unresolved issues, adapt measures to evolving contexts, and strengthen long-term ownership among involved actors [32, 39]. Embedding participation across the policy cycle may therefore enhance both the sustainability and impact of dementia strategies at the regional level (41).

The present study further illustrates that combining participatory priority-setting with subsequent policy consultation may support the transition from strategy development to implementation. Ensuring compatibility with existing governance structures while preserving priorities identified by interest-holders may be particularly important in regional policy contexts [32, 40].

Although this study focused on a specific federal state in Germany, the participatory approach combining Delphi surveys, deliberative dialogues, and policy alignment is applicable to other regional or national contexts. Regions facing demographic change, workforce shortages, or heterogeneous care structures may adopt similar structured consensus procedures to systematically collect and aggregate diverse perspectives. In the present study, no formal weighting of different participant groups was applied; assessments were aggregated across respondents using predefined consensus criteria. Future applications of this model may therefore consider whether stratified analyses or differential weighting mechanisms are appropriate to ensure balanced representation of professional and lived experiences. By explicitly documenting both consensus and non-consensus objectives, the approach enhances transparency in priority-setting and provides a replicable model for evidence-informed and participatory dementia strategy development.

## Conclusion

The development of a dementia strategy at the level of a federal state in Germany demonstrates how structured participatory methods can support transparent and inclusive priority-setting in complex health policy environments. The study generated consensus on the majority of objectives considered necessary to improve dementia care and quality of life for people living with dementia and their families in Saxony-Anhalt. Objectives for which no consensus was achieved nevertheless provided important insights into areas of ongoing debate and were further explored through deliberative dialogue. By combining formal consensus procedures with deliberative exchange and explicit documentation of both agreement and disagreement, diverse professional and lived perspectives were systematically integrated into a coherent strategic framework.

The resulting Dementia Strategy provides a foundation for future action to improve the situation of people living with dementia and their families in Saxony-Anhalt. However, the development of a strategy represents only an initial step. Meaningful improvements in care and quality of life will depend on the successful translation of strategic objectives into concrete measures, sustained political commitment, intersectoral collaboration, and the availability of adequate resources. The subsequent development of an implementation strategy will be essential to operationalize the identified objectives and support their translation into practice. The early involvement of relevant interest-holders throughout the development process may facilitate subsequent implementation by fostering ownership, cooperation, and shared responsibility for future action.

Beyond the specific regional context, this study illustrates how consensus-oriented and participatory approaches can strengthen legitimacy, traceability, and evidence-informed decision-making in dementia policy. The model may therefore inform strategy development in other regions and health systems facing demographic change, workforce pressures, and heterogeneous care structures.

## Supplementary Information

Below is the link to the electronic supplementary material.

Supplementary Material 1

Supplementary Material 2

Supplementary Material 3

Supplementary Material 4

## Data Availability

The datasets used and analysed during the current study are available from the corresponding author on reasonable request.
