# Supplementary material for: The development process of a regional dementia strategy: a Delphi study and deliberative dialogues
Source: BMC Health Serv Res. 2026 Sep 22;26:1301. doi: 10.1186/s12913-026-15595-8 (PMC13602664; doi:10.1186/s12913-026-15595-8)
Supplement: Supplementary file 1 [file 12913_2026_15595_MOESM1_ESM.docx]

**Additional file 1: Delphi questionnaire – Round 1**

English version of the questionnaire used in the first Delphi round to assess the relevance of proposed strategic objectives for the regional dementia strategy.

**Dementia Strategy of the federal state of Saxony-Anhalt –**

**Identifying objectives for a good quality of life for people living with dementia and their families**

**Questionnaire**

Dear Participant,

Thank you very much for your willingness to take part in this study on identifying objectives for the dementia strategy of Saxony-Anhalt.

Please take the time to carefully read and respond to the statements in the questionnaire. For the success of the study, it is important that you evaluate all statements honestly and according to your personal assessment.

If you have any questions, please do not hesitate to contact us:

**Dr. Anja Bieber**, Phone: +49 345 557-4427, Email: [anja.bieber@uk-halle.de](mailto:anja.bieber@uk-halle.de)

or

**Christine Schiller**, Phone: +49 345 557-1203, Email: [christine.schiller@uk-halle.de](mailto:christine.schiller@uk-halle.de)

Please return the completed questionnaire to:

**Martin Luther University Halle-Wittenberg**

**Faculty of Medicine**

**Institute for Health, Midwifery and Nursing Science**

**State Competence Center for Dementia**

**Attn.: Dr. Anja Bieber**

**Magdeburger Str. 8**

**06112 Halle (Saale), Germany**

**Personal information**

At this point, we would like to ask you to provide some information about yourself and your professional background.

**Please do not include your name, date of birth, or contact information on this questionnaire.**

| 1. **Access** (Select all that apply) | |
| --- | --- |
| I am responding as … | □ an individual.  □ an organization. |
| I am … | □ a person living with dementia.  □ an informal caregiver.  □ a professional working with people living with dementia and/or their informal caregivers.  □ in a professional role responsible for care and/or medical systems (e.g., insurance representatives, policymakers)  □ a volunteer in the field of dementia  □ not directly involved with people living with dementia or their families. |
| 2. **Employment** (Select all that apply) | |
| I … | □ work in healthcare or social services.  □ work in public administration.  □ work in government or politics.  □ work for a health or long-term care insurance organisation.  □ work in education.  □ work in research.  □ work in the nonprofit or community sector (e.g., church, police, fire department).  □ volunteer (e.g., visiting or support services).  □ am not currently employed.  □ work in another field: _____________________________________________ |
| 3. **Where you live** | |
| I live in … | □ Altmarkkreis Salzwedel.  □ Anhalt-Bitterfeld.  □ Börde.  □ Burgenlandkreis.  □ Dessau-Roßlau.  □ Halle (Saale).  □ Harz.  □ Jerichower Land.  □ Magdeburg.  □ Mansfeld-Südharz.  □ Saalekreis.  □ Salzlandkreis.  □ Stendal.  □ Wittenberg. |
| My area is … | □ rural or mostly rural.  □ urban or mostly urban. |

**Objectives for high-quality dementia care in Saxony-Anhalt**

The following statements describe objectives relevant to the care of people living with dementia.

Each statement is accompanied by a scale from 1 (not at all important) to 7 (extremely important). Please select the number that best reflects your assessment of the importance of each objective for the state of Saxony-Anhalt. If you are unable to assess a particular item, you may select “No response.”

At the end of each section, you may add additional objectives and measures that you consider important for Saxony-Anhalt.

You may also provide comments on individual statements at the end of each section. Please indicate the number of the statement you are referring to.

| **1.** | **Social participation of people living with dementia** | | | | | | | | | | |
| --- | --- | --- | --- | --- | --- | --- | --- | --- | --- | --- | --- |
| **1.1.** | **Social and living environments for people living with dementia** | | | | | | | | | | |
| **How important do you consider the following?**  (1 = Not at all important; 7 = Extremely important) | | 1 | 2 | | 3 | | 4 | 5 | 6 | 7 | No response |
| 1.1.1 | There are designated contact persons for the concerns of people living with dementia at district and municipal levels. | □ | | □ | | □ | □ | □ | □ | □ | □ |
| 1.1.2 | Municipalities have dementia-friendly^[1]^ neighborhood and village development concepts. | □ | | □ | | □ | □ | □ | □ | □ | □ |
| 1.1.3 | People living with dementia and their informal caregivers are involved in municipal or neighborhood planning processes. | □ | | □ | | □ | □ | □ | □ | □ | □ |
| 1.1.4 | Municipalities and neighborhoods provide dementia-friendly public spaces. | □ | | □ | | □ | □ | □ | □ | □ | □ |
| 1.1.5 | Housing advisory services capable of providing dementia-friendly counseling are available. | □ | | □ | | □ | □ | □ | □ | □ | □ |
| 1.1.6 | Counseling services for the needs-based establishment of shared housing arrangements for people living with dementia are available. | □ | | □ | | □ | □ | □ | □ | □ | □ |
| 1.1.7 | Nursing homes are increasingly integrated into neighborhood life. | □ | | □ | | □ | □ | □ | □ | □ | □ |
| **1.2.** | **Dementia networks** | | | | | | | | | | |
| **How important do you consider the following?**  (1 = Not at all important; 7 = Extremely important) | | 1 | | 2 | | 3 | 4 | 5 | 6 | 7 | No response |
| 1.2.1 | A permanent dementia competence center is established for the federal state of Saxony-Anhalt. | □ | | □ | | □ | □ | □ | □ | □ | □ |
| 1.2.2 | The dementia competence center provides advisory support to municipal contact persons and local dementia networks. | □ | | □ | | □ | □ | □ | □ | □ | □ |
| 1.2.3 | The dementia competence center coordinates the implementation of the Dementia Strategy of Saxony-Anhalt. | □ | | □ | | □ | □ | □ | □ | □ | □ |
| 1.2.4 | Local dementia networks receive increased funding. | □ | | □ | | □ | □ | □ | □ | □ | □ |
| 1.2.5 | Networking among dementia networks is promoted. | □ | | □ | | □ | □ | □ | □ | □ | □ |
| 1.2.6 | Local dementia networks receive advisory support from higher-level institutions. | □ | | □ | | □ | □ | □ | □ | □ | □ |

| **1.3.** | **Mobility of people living with dementia** | | | | | | | | |
| --- | --- | --- | --- | --- | --- | --- | --- | --- | --- |
| **How important do you consider the following?**  (1 = Not at all important; 7 = Extremely important) | | 1 | 2 | 3 | 4 | 5 | 6 | 7 | No response |
| 1.3.1 | Municipalities have mobility concepts tailored to the needs of people living with dementia. | □ | □ | □ | □ | □ | □ | □ | □ |
| 1.3.2 | Public transport staff receive training on dementia. | □ | □ | □ | □ | □ | □ | □ | □ |
| 1.3.3 | People living with dementia and/or their informal caregivers receive enhanced information on applying for a disability parking permit. | □ | □ | □ | □ | □ | □ | □ | □ |
| **1.4.** | **Cultural, educational, and leisure activities for people living with dementia** | | | | | | | | |
| **How important do you consider the following?**  (1 = Not at all important; 7 = Extremely important) | | 1 | 2 | 3 | 4 | 5 | 6 | 7 | No response |
| 1.4.1 | Cultural, educational, and sports facilities enable the participation of people living with dementia in regular programs. | □ | □ | □ | □ | □ | □ | □ | □ |
| 1.4.2 | Specific programs for people living with dementia are developed in the fields of culture, education, and sport. | □ | □ | □ | □ | □ | □ | □ | □ |
| 1.4.3 | Relevant interest-holders in culture, education, and sport are informed about and sensitized to dementia. | □ | □ | □ | □ | □ | □ | □ | □ |
| 1.4.4 | Interest-holders in culture, education, and sport receive guidance on promoting the cultural participation of people living with dementia. | □ | □ | □ | □ | □ | □ | □ | □ |
| **1.5.** | **Digital participation of people living with dementia** | | | | | | | | |
| **How important do you consider the following?**  (1 = Not at all important; 7 = Extremely important) | | 1 | 2 | 3 | 4 | 5 | 6 | 7 | No response |
| 1.5.1 | Digital participation of people living with dementia is improved. | □ | □ | □ | □ | □ | □ | □ | □ |
| 1.5.2 | Health care professionals are trained in the use of digital technologies in dementia care. | □ | □ | □ | □ | □ | □ | □ | □ |
| 1.5.3 | The development of technologies and digital content for people living with dementia is promoted. | □ | □ | □ | □ | □ | □ | □ | □ |
| 1.5.4 | Measures are taken to ensure that people living with dementia have access to digital resources. | □ | □ | □ | □ | □ | □ | □ | □ |
| 1.5.5 | Digital technologies support the social interaction and communication of people living with dementia. | □ | □ | □ | □ | □ | □ | □ | □ |
| **1.6.** | **Raising awareness about dementia** | | | | | | | | |
| **How important do you consider the following?**  (1 = Not at all important; 7 = Extremely important) | | 1 | 2 | 3 | 4 | 5 | 6 | 7 | No response |
| 1.6.1 | Around World Alzheimer’s Day, a Dementia Awareness Week featuring public outreach activities takes place in Saxony-Anhalt. | □ | □ | □ | □ | □ | □ | □ | □ |
| 1.6.2 | Local dementia networks conduct or support awareness-raising campaigns. | □ | □ | □ | □ | □ | □ | □ | □ |
| 1.6.3 | Awareness-raising campaigns on dementia are expanded through social media activities. | □ | □ | □ | □ | □ | □ | □ | □ |
| 1.6.4 | Children and adolescents are sensitized to dementia in an age-appropriate manner. | □ | □ | □ | □ | □ | □ | □ | □ |
| 1.6.5 | Political decision-makers are sensitized to and trained on dementia. | □ | □ | □ | □ | □ | □ | □ | □ |
| 1.6.6 | Religious organisations are trained in delivering dementia-friendly worship services and events. | □ | □ | □ | □ | □ | □ | □ | □ |
| 1.6.7 | Pastoral caregivers are trained on dementia. | □ | □ | □ | □ | □ | □ | □ | □ |

**What additional aspects related to social participation of people living with dementia are important to you?**

**Do you have any comments on one or more items in the section on social participation of people living with dementia?** (Please indicate the item number(s).)

| **2.** | **Nursing and medical care for people living with dementia** | | | | | | | | |
| --- | --- | --- | --- | --- | --- | --- | --- | --- | --- |
| **2.1.** | **Participation in medical and nursing care** | | | | | | | | |
| **How important do you consider the following?**  (1 = Not at all important; 7 = Extremely important) | | 1 | 2 | 3 | 4 | 5 | 6 | 7 | No response |
| 2.1.1 | Informal caregivers of people living with dementia are involved in the further development of care and support services. | □ | □ | □ | □ | □ | □ | □ | □ |
| 2.1.2 | Greater consideration is given to the individual needs of people living with dementia and their informal caregivers in care needs assessments. | □ | □ | □ | □ | □ | □ | □ | □ |
| **2.2.** | **Dementia-friendly design and organization of care and service facilities** | | | | | | | | |
| **How important do you consider the following?**  (1 = Not at all important; 7 = Extremely important) | | 1 | 2 | 3 | 4 | 5 | 6 | 7 | No response |
| 2.2.1 | Care and service facilities are designed in a dementia-friendly manner. | □ | □ | □ | □ | □ | □ | □ | □ |
| 2.2.2 | Dementia-specific expertise is available in care and service facilities. | □ | □ | □ | □ | □ | □ | □ | □ |
| 2.2.3 | All staff working in care and service facilities receive basic dementia-specific training. | □ | □ | □ | □ | □ | □ | □ | □ |
| 2.2.4 | Day care services are expanded. | □ | □ | □ | □ | □ | □ | □ | □ |
| 2.2.5 | Opening hours of day care facilities are made more flexible. | □ | □ | □ | □ | □ | □ | □ | □ |
| 2.2.6 | Live-in care workers in private households are more strongly integrated into the care of people living with dementia. | □ | □ | □ | □ | □ | □ | □ | □ |
| 2.2.7 | The needs of people living with dementia are given greater consideration in (emergency) hospital care. | □ | □ | □ | □ | □ | □ | □ | □ |
| 2.2.8 | Prevention and treatment of delirium in hospitals receive increased attention. | □ | □ | □ | □ | □ | □ | □ | □ |
| **2.3.** | **Medical care for people living with dementia** | | | | | | | | |
| **How important do you consider the following?**  (1 = Not at all important; 7 = Extremely important) | | 1 | 2 | 3 | 4 | 5 | 6 | 7 | No response |
| 2.3.1 | Recognized clinical guidelines for the treatment of people living with dementia are more strongly implemented in practice. | □ | □ | □ | □ | □ | □ | □ | □ |
| 2.3.2 | Digital health care services addressing the needs of people living with dementia (e.g., video consultations) are expanded. | □ | □ | □ | □ | □ | □ | □ | □ |
| 2.3.3 | The State Medical Association establishes the position of a dementia commissioner. | □ | □ | □ | □ | □ | □ | □ | □ |
| 2.3.4 | Recommendations for cross-sectoral emergency^[2]^ care for people living with dementia are available. | □ | □ | □ | □ | □ | □ | □ | □ |
| **How important do you consider the following?**  (1 = Not at all important; 7 = Extremely important) | | 1 | 2 | 3 | 4 | 5 | 6 | 7 | No response |
| 2.3.5 | Quality criteria for coordination between general practitioners and specialists in dementia care are available in Saxony-Anhalt. | □ | □ | □ | □ | □ | □ | □ | □ |

**What additional aspects related to the nursing and medical care of people living with dementia are important to you?**

**Do you have any comments on one or more items in the section on nursing and medical care of people living with dementia?** (Please indicate the item number(s).)

| **3.** | **Support for people living with dementia and their informal caregivers** | | | | | | | | |
| --- | --- | --- | --- | --- | --- | --- | --- | --- | --- |
| **3.1.** | **Counseling and support for people living with dementia and their informal caregivers** | | | | | | | | |
| **How important do you consider the following?**  (1 = Not at all important; 7 = Extremely important) | | 1 | 2 | 3 | 4 | 5 | 6 | 7 | No response |
| 3.1.1 | Community-based, dementia-specific counseling structures are expanded at the municipal level. | □ | □ | □ | □ | □ | □ | □ | □ |
| 3.1.2 | People living with dementia and their informal caregivers receive continuous local support. | □ | □ | □ | □ | □ | □ | □ | □ |
| 3.1.3 | Employment of people living with young-onset dementia is promoted. | □ | □ | □ | □ | □ | □ | □ | □ |
| 3.1.4 | Easily accessible counseling services for people living with dementia of working age are established. | □ | □ | □ | □ | □ | □ | □ | □ |
| 3.1.5 | People living with dementia receive improved support in their workplace. | □ | □ | □ | □ | □ | □ | □ | □ |
| **3.2.** | **Counseling on legal issues related to dementia** | | | | | | | | |
| **How important do you consider the following?**  (1 = Not at all important; 7 = Extremely important) | | 1 | 2 | 3 | 4 | 5 | 6 | 7 | No response |
| 3.2.1 | Dementia counseling services establish networks with legal counseling services and guardianship associations. | □ | □ | □ | □ | □ | □ | □ | □ |
| 3.2.2 | Legal guardians and guardianship judges receive training on dementia. | □ | □ | □ | □ | □ | □ | □ | □ |
| **3.3.** | **Reconciling caregiving and employment** | | | | | | | | |
| **How important do you consider the following?**  (1 = Not at all important; 7 = Extremely important) | | 1 | 2 | 3 | 4 | 5 | 6 | 7 | No response |
| 3.3.1 | The reconciliation of caregiving and employment for informal caregivers of people living with dementia is improved. | □ | □ | □ | □ | □ | □ | □ | □ |
| 3.3.2 | Informal caregivers of people living with dementia receive improved support in their workplace. | □ | □ | □ | □ | □ | □ | □ | □ |

| **3.4.** | **Culturally responsive care and counseling** | | | | | | | | |
| --- | --- | --- | --- | --- | --- | --- | --- | --- | --- |
| **How important do you consider the following?**  (1 = Not at all important; 7 = Extremely important) | | 1 | 2 | 3 | 4 | 5 | 6 | 7 | No response |
| 3.4.1 | Dementia counseling services collaborate with other culturally sensitive organizations and associations for people living with dementia with a migration background. | □ | □ | □ | □ | □ | □ | □ | □ |
| 3.4.2 | Care counseling centers provide culturally sensitive information and counseling on dementia. | □ | □ | □ | □ | □ | □ | □ | □ |
| **3.5.** | **Self-help and voluntary support** | | | | | | | | |
| **How important do you consider the following?**  (1 = Not at all important; 7 = Extremely important) | | 1 | 2 | 3 | 4 | 5 | 6 | 7 | No response |
| 3.5.1 | Voluntary engagement in the field of dementia is professionally coordinated. | □ | □ | □ | □ | □ | □ | □ | □ |
| 3.5.2 | Voluntary visiting and accompaniment services are expanded. | □ | □ | □ | □ | □ | □ | □ | □ |
| 3.5.3 | Neighborhood support initiatives are strengthened. | □ | □ | □ | □ | □ | □ | □ | □ |
| **3.6.** | **Training and information for people living with dementia and their informal caregivers** | | | | | | | | |
| **How important do you consider the following?**  (1 = Not at all important; 7 = Extremely important) | | 1 | 2 | 3 | 4 | 5 | 6 | 7 | No response |
| 3.6.1 | Dementia-specific caregiving courses and individualized in-home training programs are specifically promoted. | □ | □ | □ | □ | □ | □ | □ | □ |
| 3.6.2 | Information on dementia-specific caregiving courses and individualized in-home training programs is intensified. | □ | □ | □ | □ | □ | □ | □ | □ |
| 3.6.3 | Care services for the person living with dementia are provided when informal caregivers participate in caregiving courses. | □ | □ | □ | □ | □ | □ | □ | □ |
| 3.6.4 | Training programs for people living with dementia are developed and implemented. | □ | □ | □ | □ | □ | □ | □ | □ |
| **3.7.** | **Prevention and rehabilitation for people living with dementia and their informal caregivers** | | | | | | | | |
| **How important do you consider the following?**  (1 = Not at all important; 7 = Extremely important) | | 1 | 2 | 3 | 4 | 5 | 6 | 7 | No response |
| 3.7.1 | Preventive home visits^[3]^ for people living with dementia are carried out under municipal responsibility. | □ | □ | □ | □ | □ | □ | □ | □ |
| 3.7.2 | Rehabilitation programs for people living with dementia are expanded. | □ | □ | □ | □ | □ | □ | □ | □ |
| 3.7.3 | Rehabilitation for people living with dementia is routinely offered in the home setting. | □ | □ | □ | □ | □ | □ | □ | □ |
| 3.7.5 | Prevention, support, and rehabilitation programs for informal caregivers of people living with dementia are expanded. | □ | □ | □ | □ | □ | □ | □ | □ |
| 3.7.6 | Rehabilitation programs for informal caregivers include simultaneous care for their relatives with dementia. | □ | □ | □ | □ | □ | □ | □ | □ |
| 3.7.7 | Rehabilitation services are further developed according to dementia-friendly criteria. | □ | □ | □ | □ | □ | □ | □ | □ |
| 3.7.8 | Supervised vacations for people living with dementia and their informal caregivers are established and promoted. | □ | □ | □ | □ | □ | □ | □ | □ |
| 3.7.9 | Access to psychotherapeutic counseling for informal caregivers of people living with dementia is improved. | □ | □ | □ | □ | □ | □ | □ | □ |

| **3.8.** | **Palliative care for people living with dementia** | | | | | | | | |
| --- | --- | --- | --- | --- | --- | --- | --- | --- | --- |
| **How important do you consider the following?**  (1 = Not at all important; 7 = Extremely important) | | 1 | 2 | 3 | 4 | 5 | 6 | 7 | No response |
| 3.8.1 | Staff directly involved in the care and support of people living with dementia receive training on dementia and end-of-life care. | □ | □ | □ | □ | □ | □ | □ | □ |
| 3.8.2 | Staff in hospice and palliative care services for people living with dementia have access to supervision^[4]^. | □ | □ | □ | □ | □ | □ | □ | □ |
| 3.8.3 | Volunteer hospice workers receive dementia-specific training. | □ | □ | □ | □ | □ | □ | □ | □ |
| 3.8.4 | Examples of good practice in hospice and palliative care for people living with dementia are published and disseminated. | □ | □ | □ | □ | □ | □ | □ | □ |

**What additional aspects related to supporting people living with dementia and their family members are important to you?**

**Do you have any comments on one or more items in the section on supporting people living with dementia and their family members?** (Please indicate the item number(s).)

| **4.** | **Dementia research** | | | | | | | | |
| --- | --- | --- | --- | --- | --- | --- | --- | --- | --- |
| **4.1.** | **Involvement of people living with dementia and their informal and family caregivers** | | | | | | | | |
| **How important do you consider the following?**  (1 = Not at all important; 7 = Extremely important) | | 1 | 2 | 3 | 4 | 5 | 6 | 7 | No response |
| 4.1.1 | People living with dementia and their informal caregivers are able to participate in research projects relevant to them. | □ | □ | □ | □ | □ | □ | □ | □ |
| 4.1.2 | Procedures for involving people living with dementia in research projects are further developed. | □ | □ | □ | □ | □ | □ | □ | □ |
| **4.2.** | **Networking and strengthening dementia research in Saxony-Anhalt** | | | | | | | | |
| **How important do you consider the following?**  (1 = Not at all important; 7 = Extremely important) | | 1 | 2 | 3 | 4 | 5 | 6 | 7 | No response |
| 4.2.1 | Saxony-Anhalt establishes a research network on dementia care and support. | □ | □ | □ | □ | □ | □ | □ | □ |
| 4.2.2 | Routine data^[5]^ from dementia care and support are more accessible for research projects. | □ | □ | □ | □ | □ | □ | □ | □ |

| **4.3.** | **Research priority areas** | | | | | | | | |
| --- | --- | --- | --- | --- | --- | --- | --- | --- | --- |
| **How important do you consider the following?**  (1 = Not at all important; 7 = Extremely important) | | 1 | 2 | 3 | 4 | 5 | 6 | 7 | No response |
| 4.3.1 | The development and implementation of evidence-based^[6]^ concepts for care and support in dementia are promoted. | □ | □ | □ | □ | □ | □ | □ | □ |
| 4.3.2 | The development and implementation of evidence-based concepts for secondary and tertiary prevention^[7]^ in dementia are promoted. | □ | □ | □ | □ | □ | □ | □ | □ |
| 4.3.3 | The everyday life contexts^[8]^ of people living with dementia are increasingly explored in research. | □ | □ | □ | □ | □ | □ | □ | □ |
| 4.3.4 | Research increasingly addresses the potential of digitalization in dementia care and support. | □ | □ | □ | □ | □ | □ | □ | □ |
| 4.3.5 | The situation of informal caregivers is given greater consideration in research on dementia care and support. | □ | □ | □ | □ | □ | □ | □ | □ |
| **4.4.** | **Translation of research evidence into practice** | | | | | | | | |
| **How important do you consider the following?**  (1 = Not at all important; 7 = Extremely important) | | 1 | 2 | 3 | 4 | 5 | 6 | 7 | No response |
| 4.4.1 | Exchange between research and practice institutions on dementia care and support is promoted. | □ | □ | □ | □ | □ | □ | □ | □ |
| 4.4.2 | The transfer of evidence-based care concepts in dementia into practice is promoted. | □ | □ | □ | □ | □ | □ | □ | □ |

**What additional aspects related to dementia research are important to you?**

**Do you have any comments on one or more items in the section on dementia research?** (Please indicate the item number(s).)

**Thank you for your participation.**

1. **Dementia-friendly** refers to recognizing the needs and expectations of people living with dementia and integrating them into the design of care structures and processes.
2. **Cross-sector emergency** care refers to coordinated service delivery in which outpatient emergency practices (contracted physicians) and hospital emergency departments in a region provide aligned and integrated care.
3. **Preventive home visits** are intended to provide older adults with information at an early stage so they can take preventive measures and maintain an independent life into old age.
4. **Supervision** is a form of professional consultation designed to encourage reflection on one’s own practice and to ensure and improve the quality of professional work.
5. **Routine data** are information (e.g., age, gender, diagnoses) routinely collected by health and long-term care insurance providers or pension and accident insurance agencies in the course of service provision, for example in connection with services provided by care providers, physicians, and therapists.
6. **Evidence-based:** based on robust findings from scientific studies.
7. **Secondary prevention:** prevention in the early stages of a disease (e.g., screening and early detection measures); **Tertiary prevention:** prevention in the context of an existing disease (e.g., education and training programs)
8. E.g., research on psychosocial factors, socioeconomic inequalities, and societal perceptions of aging.
