# Supplementary material for: The development process of a regional dementia strategy: a Delphi study and deliberative dialogues
Source: BMC Health Serv Res. 2026 Sep 22;26:1301. doi: 10.1186/s12913-026-15595-8 (PMC13602664; doi:10.1186/s12913-026-15595-8)
Supplement: Supplementary file 2 [file 12913_2026_15595_MOESM2_ESM.docx]

**Additional file 2: Delphi questionnaire – Round 2**

English version of the questionnaire used in the second Delphi round to assess the relevance of proposed strategic objectives for the regional dementia strategy.

**Dementia Strategy of the federal state of Saxony-Anhalt –**

**Identifying objectives for a good quality of life for people living with dementia and their families**

**Questionnaire for Survey Round 2**

Dear Participant,

Thank you for your participation in the first round of this study on identifying objectives and measures for the dementia strategy of Saxony-Anhalt. With your valuable input, consensus has already been reached on a number of objectives and measures.

This second round includes those objectives and measures for which no consensus has yet been achieved, as well as additional suggestions from the first survey. We kindly invite you to participate in this second round.

Please take the time to carefully read and respond to the questionnaire items. The success of the study depends on your honest evaluation of each item based on your personal assessment.

If you have any questions, please do not hesitate to contact us:

**Dr. Anja Bieber**, Phone: +49 345 557-4427, Email: [anja.bieber@uk-halle.de](mailto:anja.bieber@uk-halle.de)

or

**Christine Schiller**, Phone: +49 345 557-1203, Email: [christine.schiller@uk-halle.de](mailto:christine.schiller@uk-halle.de)

Please return the completed questionnaire to:

**Martin Luther University Halle-Wittenberg**

**Faculty of Medicine**

**Institute for Health, Midwifery and Nursing Science**

**State Competence Center for Dementia**

**Attn.: Dr. Anja Bieber**

**Magdeburger Str. 8**

**06112 Halle (Saale), Germany**

**Personal information**

At this point, we would like to ask you to provide some information about yourself and your professional background. This information will be used to match your responses with those from the first survey round.

**Please do not include your name, date of birth, or contact information on this questionnaire.**

| 1. **Access** (Select all that apply) | |
| --- | --- |
| I am responding as … | □ an individual.  □ an organization. |
| I am … | □ a person living with dementia.  □ an informal caregiver.  □ a professional working with people living with dementia and/or their informal caregivers.  □ in a professional role responsible for care and/or medical systems (e.g., insurance representatives, policymakers)  □ a volunteer in the field of dementia  □ not directly involved with people living with dementia or their families. |
| 2. **Employment** (Select all that apply) | |
| I … | □ work in healthcare or social services.  □ work in public administration.  □ work in government or politics.  □ work for a health or long-term care insurance organisation.  □ work in education.  □ work in research.  □ work in the nonprofit or community sector (e.g., church, police, fire department).  □ volunteer (e.g., visiting or support services).  □ am not currently employed.  □ work in another field: _____________________________________________ |
| 3. **Where you live** | |
| I live in … | □ Altmarkkreis Salzwedel.  □ Anhalt-Bitterfeld.  □ Börde.  □ Burgenlandkreis.  □ Dessau-Roßlau.  □ Halle (Saale).  □ Harz.  □ Jerichower Land.  □ Magdeburg.  □ Mansfeld-Südharz.  □ Saalekreis.  □ Salzlandkreis.  □ Stendal.  □ Wittenberg. |
| My area is … | □ rural or mostly rural.  □ urban or mostly urban. |

**Objectives for high-quality dementia care in Saxony-Anhalt**

The following statements describe objectives relevant to the care of people living with dementia.

Each statement is accompanied by a scale from 1 (not at all important) to 7 (extremely important). Please select the number that best reflects your assessment of the importance of each objective for the state of Saxony-Anhalt. If you are unable to assess a particular item, you may select “No response.”

You may also provide comments on individual statements at the end of each section. Please indicate the number of the statement you are referring to.

| **1.** | **Social participation of people living with dementia** | | | | | | | | | | |
| --- | --- | --- | --- | --- | --- | --- | --- | --- | --- | --- | --- |
| **1.1.** | **Social and living environments for people living with dementia** | | | | | | | | | | |
| **How important do you consider the following?**  (1 = Not at all important; 7 = Extremely important) | | 1 | 2 | | 3 | | 4 | 5 | 6 | 7 | No response |
| 1.1.1 | Municipalities have dementia-friendly^[1]^ neighborhood and village development concepts. | □ | | □ | | □ | □ | □ | □ | □ | □ |
| 1.1.2 | People living with dementia and their informal caregivers are involved in municipal or neighborhood planning processes. | □ | | □ | | □ | □ | □ | □ | □ | □ |
| 1.1.3 | Municipalities and neighborhoods provide dementia-friendly public spaces^[2]^. | □ | | □ | | □ | □ | □ | □ | □ | □ |
| **1.2.** | **Dementia networks** | | | | | | | | | | |
| **How important do you consider the following?**  (1 = Not at all important; 7 = Extremely important) | | 1 | | 2 | | 3 | 4 | 5 | 6 | 7 | No response |
| 1.2.1 | Local dementia networks receive advisory support from higher-level institutions. | □ | | □ | | □ | □ | □ | □ | □ | □ |
| **1.3.** | **Mobility of people living with dementia** | | | | | | | | | | |
| **How important do you consider the following?**  (1 = Not at all important; 7 = Extremely important) | | 1 | | 2 | | 3 | 4 | 5 | 6 | 7 | No response |
| 1.3.1 | Municipalities have mobility concepts tailored to the needs of people living with dementia. | □ | | □ | | □ | □ | □ | □ | □ | □ |
| 1.3.2 | Public transport staff receive training on dementia. | □ | | □ | | □ | □ | □ | □ | □ | □ |
| 1.3.3 | People living with dementia and/or their informal caregivers receive enhanced information on applying for a disability parking permit. | □ | | □ | | □ | □ | □ | □ | □ | □ |
| **1.4.** | **Cultural, educational, and leisure activities for people living with dementia** | | | | | | | | | | |
| **How important do you consider the following?**  (1 = Not at all important; 7 = Extremely important) | | 1 | | 2 | | 3 | 4 | 5 | 6 | 7 | No response |
| 1.4.1 | Cultural, educational, and sports facilities enable the participation of people living with dementia in regular programs. | □ | | □ | | □ | □ | □ | □ | □ | □ |
| 1.4.2 | Specific programs for people living with dementia are developed in the fields of culture, education, and sport. | □ | | □ | | □ | □ | □ | □ | □ | □ |
| 1.4.3 | Relevant interest-holders in culture, education, and sport are informed about and sensitized to dementia. | □ | | □ | | □ | □ | □ | □ | □ | □ |
| 1.4.4 | Interest-holders in culture, education, and sport receive guidance on promoting the cultural participation of people living with dementia. | □ | | □ | | □ | □ | □ | □ | □ | □ |
| **1.5.** | **Digital participation^[3]^ of people living with dementia** | | | | | | | | | | |
| **How important do you consider the following?**  (1 = Not at all important; 7 = Extremely important) | | 1 | | 2 | | 3 | 4 | 5 | 6 | 7 | No response |
| 1.5.1 | Digital participation of people living with dementia is improved. | □ | | □ | | □ | □ | □ | □ | □ | □ |
| 1.5.2 | Health care professionals are trained in the use of digital technologies in dementia care. | □ | | □ | | □ | □ | □ | □ | □ | □ |
| 1.5.3 | The development of technologies and digital content for people living with dementia is promoted. | □ | | □ | | □ | □ | □ | □ | □ | □ |
| 1.5.4 | Measures are taken to ensure that people living with dementia have access to digital resources. | □ | | □ | | □ | □ | □ | □ | □ | □ |
| 1.5.5 | Digital technologies support the social interaction and communication of people living with dementia. | □ | | □ | | □ | □ | □ | □ | □ | □ |
| **1.6.** | **Raising awareness about dementia** | | | | | | | | | | |
| **How important do you consider the following?**  (1 = Not at all important; 7 = Extremely important) | | 1 | | 2 | | 3 | 4 | 5 | 6 | 7 | No response |
| 1.6.1 | Around World Alzheimer’s Day, a Dementia Awareness Week featuring public outreach activities takes place in Saxony-Anhalt. | □ | | □ | | □ | □ | □ | □ | □ | □ |
| 1.6.2 | Local dementia networks conduct or support awareness-raising campaigns. | □ | | □ | | □ | □ | □ | □ | □ | □ |
| 1.6.3 | Awareness-raising campaigns on dementia are expanded through social media activities. | □ | | □ | | □ | □ | □ | □ | □ | □ |
| 1.6.4 | Children and adolescents are sensitized to dementia in an age-appropriate manner. | □ | | □ | | □ | □ | □ | □ | □ | □ |
| 1.6.5 | Religious organisations are trained in delivering dementia-friendly worship services and events. | □ | | □ | | □ | □ | □ | □ | □ | □ |
| 1.6.6 | Pastoral caregivers are trained on dementia. | □ | | □ | | □ | □ | □ | □ | □ | □ |
| 1.6.7 | Employees across all areas of everyday life (e.g., retail, restaurants, public transport) are sensitized to dementia. | □ | | □ | | □ | □ | □ | □ | □ | □ |
| **Do you have any comments on one or more items in the section on social participation of people living with dementia?** (Please indicate the item number(s).) | | | | | | | | | | | |

| **2.** | **Nursing and medical care for people living with dementia** | | | | | | | | |
| --- | --- | --- | --- | --- | --- | --- | --- | --- | --- |
| **2.1.** | **Dementia-friendly design and organization of care and service facilities** | | | | | | | | |
| **How important do you consider the following?**  (1 = Not at all important; 7 = Extremely important) | | 1 | 2 | 3 | 4 | 5 | 6 | 7 | No response |
| 2.1.1 | The expansion of alternative care models^[4]^ is promoted. | □ | □ | □ | □ | □ | □ | □ | □ |

| **2.2.** | **Medical care for people living with dementia** | | | | | | | | |
| --- | --- | --- | --- | --- | --- | --- | --- | --- | --- |
| **How important do you consider the following?**  (1 = Not at all important; 7 = Extremely important) | | 1 | 2 | 3 | 4 | 5 | 6 | 7 | No response |
| 2.2.1 | Digital health care services addressing the needs of people living with dementia (e.g., video consultations) are expanded. | □ | □ | □ | □ | □ | □ | □ | □ |
| 2.2.2 | The State Medical Association establishes the position of a dementia commissioner. | □ | □ | □ | □ | □ | □ | □ | □ |
| 2.2.3 | Recommendations for cross-sectoral emergency^[5]^ care for people living with dementia are available. | □ | □ | □ | □ | □ | □ | □ | □ |
| 2.2.4 | Quality criteria for coordination between general practitioners and specialists in dementia care^[6]^ are available in Saxony-Anhalt. | □ | □ | □ | □ | □ | □ | □ | □ |
| 2.2.5 | Medical care and treatment of people living with dementia through home visits (e.g., by qualified health professionals) are strengthened. | □ | □ | □ | □ | □ | □ | □ | □ |
| 2.2.6 | The involvement of informal caregivers in all aspects of care is improved. | □ | □ | □ | □ | □ | □ | □ | □ |
| **2.3.** | **Therapeutic services for people living with dementia** | | | | | | | | |
| **How important do you consider the following?**  (1 = Not at all important; 7 = Extremely important) | | 1 | 2 | 3 | 4 | 5 | 6 | 7 | No response |
| 2.3.1 | Therapeutic services such as occupational therapy, speech therapy, and physiotherapy are prescribed for people living with dementia. | □ | □ | □ | □ | □ | □ | □ | □ |
| 2.3.2 | Therapeutic services for people living with dementia are expanded, for example through music therapy and psychotherapy. | □ | □ | □ | □ | □ | □ | □ | □ |
| **Do you have any comments on one or more items in the section on nursing and medical care of people living with dementia?** (Please indicate the item number(s).) | | | | | | | | | |

| **3.** | **Support for people living with dementia and their informal caregivers** | | | | | | | | |
| --- | --- | --- | --- | --- | --- | --- | --- | --- | --- |
| **3.1.** | **Counseling and support for people living with dementia and their informal caregivers** | | | | | | | | |
| **How important do you consider the following?**  (1 = Not at all important; 7 = Extremely important) | | 1 | 2 | 3 | 4 | 5 | 6 | 7 | No response |
| 3.1.1 | Employment of people living with young-onset dementia is promoted. | □ | □ | □ | □ | □ | □ | □ | □ |
| 3.1.2 | People living with dementia receive improved support in their workplace. | □ | □ | □ | □ | □ | □ | □ | □ |
| **3.2.** | **Culturally responsive^[7]^ care and counseling** | | | | | | | | |
| **How important do you consider the following?**  (1 = Not at all important; 7 = Extremely important) | | 1 | 2 | 3 | 4 | 5 | 6 | 7 | No response |
| 3.2.1 | Dementia counseling services collaborate with other culturally sensitive organizations and associations for people living with dementia with a migration background. | □ | □ | □ | □ | □ | □ | □ | □ |
| 3.2.2 | Care counseling centers provide culturally sensitive information and counseling on dementia. | □ | □ | □ | □ | □ | □ | □ | □ |
| **3.3.** | **Self-help and voluntary support** | | | | | | | | |
| **How important do you consider the following?**  (1 = Not at all important; 7 = Extremely important) | | 1 | 2 | 3 | 4 | 5 | 6 | 7 | No response |
| 3.3.1 | Voluntary engagement in the field of dementia is professionally coordinated. | □ | □ | □ | □ | □ | □ | □ | □ |
| **3.4.** | **Training and information for people living with dementia and their informal caregivers** | | | | | | | | |
| **How important do you consider the following?**  (1 = Not at all important; 7 = Extremely important) | | 1 | 2 | 3 | 4 | 5 | 6 | 7 | No response |
| 3.4.1 | Training programs for people living with dementia are developed and implemented. | □ | □ | □ | □ | □ | □ | □ | □ |
| 3.4.2 | Activity materials and (state-specific) information materials are freely accessible to people living with dementia and their caregivers. | □ | □ | □ | □ | □ | □ | □ | □ |
| **3.5.** | **Prevention and rehabilitation for people living with dementia and their informal caregivers** | | | | | | | | |
| **How important do you consider the following?**  (1 = Not at all important; 7 = Extremely important) | | 1 | 2 | 3 | 4 | 5 | 6 | 7 | No response |
| 3.5.1 | Preventive home visits^[8]^ for people living with dementia are carried out under municipal responsibility. | □ | □ | □ | □ | □ | □ | □ | □ |
| 3.5.2 | Rehabilitation programs for people living with dementia are expanded. | □ | □ | □ | □ | □ | □ | □ | □ |
| 3.5.3 | Rehabilitation for people living with dementia is routinely offered in the home setting. | □ | □ | □ | □ | □ | □ | □ | □ |
| **3.6.** | **Self-help and support for informal caregivers of people living with dementia** | | | | | | | | |
| **How important do you consider the following?**  (1 = Not at all important; 7 = Extremely important) | | 1 | 2 | 3 | 4 | 5 | 6 | 7 | No response |
| 3.6.1 | (Regional) respite and recovery services for informal caregivers of people living with dementia are expanded. | □ | □ | □ | □ | □ | □ | □ | □ |
| 3.6.2 | Regional and digital exchange opportunities/platforms for and with people living with (early-stage) dementia are established across the state. | □ | □ | □ | □ | □ | □ | □ | □ |
| 3.6.3 | Self-help groups and associations focusing on dementia are promoted. | □ | □ | □ | □ | □ | □ | □ | □ |
| **Do you have any comments on one or more items in the section on supporting people living with dementia and their family members?** (Please indicate the item number(s).) | | | | | | | | | |

| **4.** | **Dementia research** | | | | | | | | |
| --- | --- | --- | --- | --- | --- | --- | --- | --- | --- |
| **4.1.** | **Involvement of people living with dementia and their informal and family caregivers** | | | | | | | | |
| **How important do you consider the following?**  (1 = Not at all important; 7 = Extremely important) | | 1 | 2 | 3 | 4 | 5 | 6 | 7 | No response |
| 4.1.1 | People living with dementia and their informal caregivers are able to participate in research projects relevant to them. | □ | □ | □ | □ | □ | □ | □ | □ |
| 4.1.2 | Procedures for involving people living with dementia in research projects are further developed. | □ | □ | □ | □ | □ | □ | □ | □ |

| **4.2.** | **Networking and strengthening dementia research in Saxony-Anhalt** | | | | | | | | |
| --- | --- | --- | --- | --- | --- | --- | --- | --- | --- |
| **How important do you consider the following?**  (1 = Not at all important; 7 = Extremely important) | | 1 | 2 | 3 | 4 | 5 | 6 | 7 | No response |
| 4.2.1 | Saxony-Anhalt establishes a research network on dementia care and support. | □ | □ | □ | □ | □ | □ | □ | □ |
| 4.2.2 | Routine data^[9]^ from dementia care and support are more accessible for research projects. | □ | □ | □ | □ | □ | □ | □ | □ |
| **4.3.** | **Research priority areas** | | | | | | | | |
| **How important do you consider the following?**  (1 = Not at all important; 7 = Extremely important) | | 1 | 2 | 3 | 4 | 5 | 6 | 7 | No response |
| 4.3.1 | The development and implementation of evidence-based^[10]^ concepts for care and support in dementia are promoted. | □ | □ | □ | □ | □ | □ | □ | □ |
| 4.3.2 | Research increasingly addresses the potential of digitalization in dementia care and support. | □ | □ | □ | □ | □ | □ | □ | □ |
| 4.3.3 | The current state of nursing and medical care for people living with dementia in Saxony-Anhalt is assessed. | □ | □ | □ | □ | □ | □ | □ | □ |
| **4.4.** | **Translation of research evidence into practice** | | | | | | | | |
| **How important do you consider the following?**  (1 = Not at all important; 7 = Extremely important) | | 1 | 2 | 3 | 4 | 5 | 6 | 7 | No response |
| 4.4.1 | Collaboration between research and practice institutions on dementia is strengthened. | □ | □ | □ | □ | □ | □ | □ | □ |
| **4.5.** | **Strengthening infrastructure in dementia research** | | | | | | | | |
| **How important do you consider the following?**  (1 = Not at all important; 7 = Extremely important) | | 1 | 2 | 3 | 4 | 5 | 6 | 7 | No response |
| 4.5.1 | The federal state promotes practice-oriented research on dementia care and support. | □ | □ | □ | □ | □ | □ | □ | □ |
| **Do you have any comments on one or more items in the section on dementia research?** (Please indicate the item number(s).) | | | | | | | | | |

**Thank you for your participation.**

1. **Dementia-friendly** refers to recognizing the needs and expectations of people living with dementia and integrating them into the design of care structures and processes.
2. **Dementia-friendly public spaces** are intended to provide people living with dementia with a safe environment in which they can navigate more easily. Key features include clear signage and wayfinding aids, accessible and well-structured pathways, appropriate use of color, quiet retreat areas, and shared spaces that support social interaction.
3. **Digital participation of people living with dementia** refers to their access to digital technologies and content, as well as their ability to use them easily. This includes adapted devices, specialized applications, individualized support, and accessible digital services. The aim is to enable people living with dementia to benefit from digitalization and to promote their independence and social participation.
4. **Alternative care models** refer to care and housing arrangements for people living with dementia outside of traditional residential (institutional) care (e.g., shared housing arrangements, care farms).
5. **Cross-sector emergency** care refers to coordinated service delivery in which outpatient emergency practices (contracted physicians) and hospital emergency departments in a region provide aligned and integrated care.
6. **Networking in primary and specialist care** refers to the coordinated collaboration between general practitioners, specialists, and other key professional groups involved in nursing care, therapy, and support services.
7. **Culturally responsive care means that the cultural, linguistic, and social backgrounds of people living with dementia are taken into account.**
8. **Preventive home visits** are intended to provide older adults with information at an early stage so they can take preventive measures and maintain an independent life into old age.
9. **Routine data** are information (e.g., age, gender, diagnoses) routinely collected by health and long-term care insurance providers or pension and accident insurance agencies in the course of service provision, for example in connection with services provided by care providers, physicians, and therapists.
10. **Evidence-based:** based on robust findings from scientific studies.
