# Supplementary material for: The development process of a regional dementia strategy: a Delphi study and deliberative dialogues
Source: BMC Health Serv Res. 2026 Sep 22;26:1301. doi: 10.1186/s12913-026-15595-8 (PMC13602664; doi:10.1186/s12913-026-15595-8)
Supplement: Supplementary file 3 [file 12913_2026_15595_MOESM3_ESM.docx]

**Additional file 3: Discussion guide for deliberative dialogues**

Structured discussion guide used in the deliberative dialogues, including predefined questions and workshop procedure.

**Introduction**

- Welcome and introduction of participants
- Explanation of the objectives of the deliberative dialogues
- Information on data collection and consent procedures

**Workshop procedure**

The deliberative dialogues followed a structured workshop format:

- Presentation of the workshop agenda
- Introduction round of participants
- Presentation of results from the Delphi survey
- Presentation of objectives without consensus and guiding questions
- Group discussion and joint reflection
- Summary of results and closing

**Discussion focus**

Participants discussed selected objectives within the thematic area of awareness-raising that had not reached consensus in the Delphi survey, as these were considered conceptually complex and required further clarification.

Discussion aimed to:

- explore reasons for disagreement
- clarify conceptual ambiguities
- identify potential reformulations or alternative perspectives

**Guiding questions**

The discussion was structured using predefined guiding questions, including:

- Which target groups should be addressed?
- Which communication channels and methods are most effective?
- Who should be responsible for implementation?
- Which structural or contextual conditions are required?

**Discussion principles**

To support a balanced and respectful exchange, the following principles were applied:

- Respect different perspectives and experiences
- Encourage participation from all participants
- Maintain a constructive and solution-oriented discussion
- Acknowledge and reflect differing opinions

**Documentation and synthesis**

- Key discussion points were documented during the deliberative dialogues
- Results were summarized and and used to refine objectives

**Closing**

- Summary of key discussion results
- Opportunity for final comments
